# Prevalence, patterns, and impact of myofascial pain in patients with head and neck cancer after cancer treatment - a single-center cross-sectional study in India

**DOI:** 10.1186/s12904-025-01745-y

**Published:** 2025-04-21

**Authors:** Aswathi Praveen, Krithika Rao, S. Gayatri, Anuja D. Damani, Arun Ghoshal, Shreya Nair, Naveena A. N. Kumar, Shirley Lewis Salins, Ananth Pai, Anshul Singh, Karthik S. Udupa, Nawaz Usman, Naveen S. Salins

**Affiliations:** 1 Department of Palliative Medicine and Supportive Care, Kasturba Medical College, Manipal Academy of Higher Education, Manipal, Karnataka 576104 India; 2 Department of Surgical Oncology, Kasturba Medical College, Manipal Academy of Higher Education, Manipal, Karnataka 576104 India; 3 Department of Radiotherapy and Oncology, Kasturba Medical College, Manipal Academy of Higher Education, Manipal, Karnataka 576104 India; 4 Department of Medical Oncology, Kasturba Medical College, Manipal Academy of Higher Education, Manipal, Karnataka 576104 India

**Keywords:** Myofascial pain syndrome, Head and neck cancer, Depression, Myofascial trigger points

## Abstract

**Purpose:**

Head and neck cancer is the seventh most prevalent cancer, with over 660,000 new cases and 325,000 annual fatalities, accounting for 30% of all cancer cases. Chronic cancer-related pain affects 15–75% of patients, with myofascial pain being especially common in those with head and neck cancers, ranging from 11.9 to 44.8%. Surgery and radiotherapy, the primary treatments for these cancers, contribute to myofascial pain development. Additionally, head and neck cancer patients face higher psychological distress, with rates up to 50%. This study estimates the prevalence, topography of the musculoskeletal group, and emotional impact of myofascial pain in terms of depression in patients three months post-treatment, emphasizing early diagnosis for improved quality of life.

**Methods:**

We conducted a time-bound cross-sectional observational study using convenience sampling of 120 patients with head and neck cancer who were post-surgery or radiotherapy over a 12-month period from April 2023 to March 2024. Data were collected using structured proforma and validated tools. Descriptive statistics summarized continuous variables, and chi-square tests compared categorical variables. Pearson correlation measured linear relationships, while regression analysis estimated the relationship between pain and explanatory variables. A two-sided p-value of < 0.05 was considered statistically significant.

**Results:**

The prevalence of myofascial pain was 68.3% (*n* = 82), though no statistically significant relationship was found between its occurrence and time since therapy (*p* > 0.05). The most affected muscle was sternocleidomastoid (55%), followed by masseter (29.2%), trapezius (25.8%), temporalis (15%), levator scapulae (8.3%), posterior cervical (5.8%), and splenius capitis (3.3%). Additionally, 75.8% (*n* = 91) of participants had depression on PHQ-9, with 85.4% (*n* = 70) with myofascial pain experiencing depressive symptoms. Pain score and depression in patients with myofascial pain were positively correlated with a value of 0.579 (p-value < 0.05).

**Conclusion:**

Myofascial pain is common in patients with head and neck cancer post-treatment with a negative impact on emotional well-being. It primarily affects the muscles involved in the neck and shoulder movements. It is important to identify early and manage the complications to enhance quality of life.

**Clinical trial registration:**

The study is registered with the Clinical Trials Registry India and the assigned registration number for this study is CTRI/2023/03/050268 on 02/03/2023.

**Supplementary Information:**

The online version contains supplementary material available at 10.1186/s12904-025-01745-y.

## Introduction

Cancer is a significant global health challenge, contributing to approximately 1 in 6 deaths worldwide, or 16.8% of all deaths [1]. Among the various forms of cancer, head and neck cancer is particularly prevalent, ranking as the seventh most common type globally, with over 660,000 new cases and around 325,000 deaths annually [1]. This incidence is expected to rise, with projections indicating a 30% increase by 2030. In India, the cancer burden is on the rise, with Global Cancer Observatory 2020 predicting a 57.5% increase in cancer cases by 2040, translating to 2.1 million new cases, of which head and neck cancer constitutes a significant portion, representing 30% of all cases [2].

Head and neck cancer encompasses malignancies that affect the oral cavity, pharynx, and larynx, most commonly originating from the mucosal epithelium [3]. Other less common sites include the salivary glands, nose, sinuses, and muscles. Squamous cell carcinoma is the predominant form, accounting for 4.5% of all global cancer diagnoses and 4.6% of cancer-related deaths [4]. Treatment for head and neck cancer is complex and depends on the stage and location of the disease [5]. Therapeutic options include single-modality treatments like surgery or radiotherapy, as well as combined modalities such as surgery with radiotherapy or chemo/immunotherapy to improve outcomes [6].

The symptom burden associated with head and neck cancer is substantial. A study conducted at a tertiary hospital in India identified common symptoms such as pain, insomnia, loss of appetite, and fatigue [7]. Other symptoms included difficulty swallowing, dry mouth, and difficulty opening the mouth, while symptoms like dyspnea, loss of taste, cough, and nausea were less frequently reported [7]. Treatment-related toxicities also affect the quality of life, with acute toxicities such as dysphagia, dermatitis, mucositis, and weight loss being common. Long-term toxicities include muscle fibrosis, osteonecrosis, and vascular complications [8].

Pain is a complex and prevalent issue in cancer, with chronic cancer-related pain affecting 15–75% of patients, depending on the type and extent of the disease [9]. Myofascial pain syndrome, a non-inflammatory musculoskeletal pain disorder characterized by muscle stiffness and trigger points, is particularly common in patients with head and neck cancer, with a prevalence ranging from 11.9 to 44.8% [10]. Cancer-directed treatments like surgery and radiotherapy cause tissue manipulation, muscle fibrosis, and chronic inflammation which could exacerbate myofascial pain [11].

In addition to physical pain, emotional distress, anxiety, and depression are common among head and neck cancer patients [12]. Psychological distress can significantly exacerbate the experience of pain and contribute to a lower quality of life. Research has shown that individuals with head and neck cancer have higher rates of psychological distress, with up to 50% experiencing significant emotional challenges like depression affecting postoperative performance status, length of hospital stay, and compliance to treatment [12]. Depressive disorders are associated with lower survival rates and disease-free status, as well as a decline in postoperative function and overall quality of life. Identifying and addressing these issues is critical to improving the overall well-being of head and neck cancer patients [13].

Given the substantial impact of myofascial pain on both physical and emotional health, early diagnosis and management are crucial for improving the quality of life in post-treatment head and neck cancer patients [14]. This study aims to estimate the point prevalence of myofascial pain in these patients, examine patterns of muscle involvement, and explore the psychological impact of this condition.

The objectives of the study were -.

1) To know the point prevalence of myofascial pain in patients with head and neck cancer using the myofascial pain diagnostic criteria for trigger points as per international consensus on the cluster of criteria for trigger point diagnosis.

2) To assess patterns of myofascial pain distribution in patients with head and neck cancers; draw its topographical representation; identify myofascial trigger points and correlate the patterns with individual/group of muscles.

3) To evaluate the impact of myofascial pain on emotional health in patients with head and neck cancer using the nine-item Patient Health Questionnaire (PHQ-9) questionnaire.

## Methods

### Study design, setting, and ethical approval

This study was a cross-sectional observational research project conducted over a period of 12 months, from April 2023 to March 2024. It involved patients with head and neck cancer who were three months post-cancer-directed treatment and presented to the oncology departments of a tertiary care hospital in Karnataka for follow-up assessment and referred to Palliative care services for symptom management and supportive care, Palliative care services is well integrated into oncology care at this center. Prior approval from the Institutional Review Board (IEC2: 493/2022) was obtained and all participants gave written informed consent during study enrolment.

### Participants

The participants for this study were selected according to well-defined inclusion and exclusion criteria. The inclusion criteria encompassed all patients who had completed cancer-directed treatments—surgery, radiotherapy, or a combination of both—at least three months prior and were aged 18 years or older, irrespective of whether they experienced pain and with a histopathological diagnosis of head and neck cancer. Only patients who provided informed consent were included in the study. Prior history for head and shoulder pain before cancer diagnosis was not exclusively assessed however patients we excluded were individuals who had received only chemotherapy or any systemic treatment or had a pre-existing diagnosis of fibromyalgia or other clinical conditions that could contribute to myofascial pain before their cancer diagnosis.

### Outcome measures

1. Recording the point prevalence of myofascial pain in patients with head and neck cancer using the myofascial pain diagnostic criteria for trigger points as per the international consensus on the cluster of criteria for trigger point diagnosis.
2. Documenting the topographical representation and patterns of specific muscle groups involved in myofascial pain syndromes in patients with head and neck cancer after assessing history and clinical examination of myofascial trigger points.
3. Understanding the impact of long-standing myofascial pain on the emotional health of patients with head and neck cancer utilizing the PHQ9 questionnaire.

### Study tools

#### **Diagnostic criteria for the myofascial trigger points based on the international consensus on diagnostic criteria and clinical considerations of myofascial trigger points** [15] (*Appendix*: − 1– *Supplementary file*)

**Numerical rating scale (NRS) for pain intensity** [16].

- A unidimensional measure of pain intensity where an individual selects a whole number between 0 and 10 that best reflects the pain intensity.
- The 11-point numeric scale from 0 representing one pain extreme (i.e., no pain) to 10 representing the other pain extreme (i.e., worst imaginable pain).
- It is easy to complete, administer, and score as the tool is valid and reliable in measuring pain intensity [17].

**Patient health questionnaire − 9 (PHQ-9)** [18] (*Appendix*: 2 and 3– *Supplementary file*).

- Patient health questionnaire is a self-administered questionnaire that is a diagnostic instrument for common mental disorders.
- PHQ-9 is a 9-item depression score, which is a reliable and valid tool for assessing depression severity [19].
- The scale is translated and validated in multiple local languages, including Kannada which was used in this study [18].
- The score ranges between 0 and 27, as each of the 9 questions is scored from 0 to 3.

### Sample size and statistical methods

We conducted a time-bound cross-sectional observational study using convenience sampling over a 12-month period from April 2023 to March 2024. The study focused on patients with head and neck cancer who were 3 months post-cancer treatment and were visiting the oncology departments of a tertiary hospital in Karnataka. We analyzed the data using Jamovi 2.5.5 software. Descriptive statistics, such as frequency, mean, and range, were used to summarize continuous variables. We used chi-square tests to compare categorical variables, Pearson correlation to measure the strength of linear relationships between variables, and a regression model to estimate the linear relationship between pain (a scalar response) and one or more explanatory variables. A two-sided p-value less than 0.05 was considered significant.

## Results

Out of 124 patients screened, 120 were included, and 4 were excluded from the study, as one declined to participate. Three patients did not have a confirmed diagnosis of head and neck cancer on histopathology. Table 1 outlines the patient characteristics.

**Table 1:** Patient characteristics

	% (*n*)
**Total patients**	100 (*n* = 120)
Males	84.2 (*n* = 101)
Females	15.8 (*n* = 19)
**Age (in years)**	
Range	24–81
Mean	55.91
Standard deviation	11.706
18–24 years	0.8 (*n* = 1)
25–44 years	16.7 (*n* = 20)
45–60 years	45 (*n* = 54)
61–75 years	35 (*n* = 42)
76–90 years	2.5 (*n* = 3)
**Occupation**	
Skilled	5.8 (*n* = 7)
Unskilled	65.8 (*n* = 79)
Unemployed	28.4 (*n* = 34)
**Comorbidity**	
No comorbidity	60.0 (*n* = 72)
Single comorbidity	21.7 (*n* = 26)
Multimorbidity	18.3 (*n* = 22)
**Head and neck cancer diagnosis site**	
Oral cavity	64.1 (*n* = 77)
Oropharynx	11.7 (*n* = 14)
Hypopharynx	10.0 (*n* = 12)
Larynx	12.5 (*n* = 15)
Others (nasopharynx/paranasal sinus/salivary glands)	1.7 (*n* = 2)
**Stage of malignancy**	
Non-metastatic	94.2 (*n* = 113)
Metastatic	5.8 (*n* = 7)
**Treatment received**	
Surgery	4.2 (*n* = 5)
Surgery + Adjuvant treatment (RT/CTRT)	57.5 (*n* = 69)
Definitive treatment (RT/CTRT)	37.5 (*n* = 45)
Palliative RT	0.8 (*n* = 1)
**Dose of CTRT**	*n* = 56
70 Gy/35#	57.1 (*n* = 32)
66 Gy/30#	3.6 (*n* = 2)
66 Gy/33#	26.8 (*n* = 15)
Others	12.5 (*n* = 7)
**Dose of RT**	*n* = 59
70 Gy/35#	15.3 (*n* = 9)
60 Gy/30#	78.0 (*n* = 46)
66 Gy/33#	5.1 (*n* = 3)
22.5 Gy/5#	1.7 (*n* = 1)
**Post-treatment period (months)**	
3–12	58.3 (*n* = 78)
13–36	29.2 (*n* = 35)
> 36	12.5 (*n* = 15)

**Table 2:** Regression analysis

Dependent variable			Pain score		
R-squared			0.391		
Adjusted R-squared			0.367		
F-statistic			16.68		
Number of observations			82		
**Variable**	**Coefficient**	**Standard error**		**P value**	**[0.025–0.975]**
Intercept	3.6982	0.538		0.000	2.626–4.770
Age	-0.0009	0.009		0.918	-0.019–0.017
Months post-treatment	-0.0102	0.004		0.010	-0.018 - -0.002
Depression score	0.1457	0.022		0.000	0.101–0.190

Pain was reported by 78.3% (*n* = 94) of patients, where 68.3% (*n* = 82) of them had myofascial pain as per the international consensus on the cluster of criteria for trigger point diagnosis. Out of the participants with myofascial pain, 72.0% (*n* = 59) experienced pain of moderate intensity, 24.4% (*n* = 20) experienced mild intensity, and 3.7% (*n* = 3) experienced severe intensity according to the numerical rating scale.

63.4% (*n* = 52) of patients with myofascial pain were in the period of 3 to 12 months post-treatment. 24.4% (*n* = 20) had myofascial pain in a period of 13–36 months post-treatment. 36 months following treatment, 12.2% (*n* = 10) experienced myofascial pain. There was no statistically significant correlation observed between these groups (p value > 0.05).

**Fig. 1:**
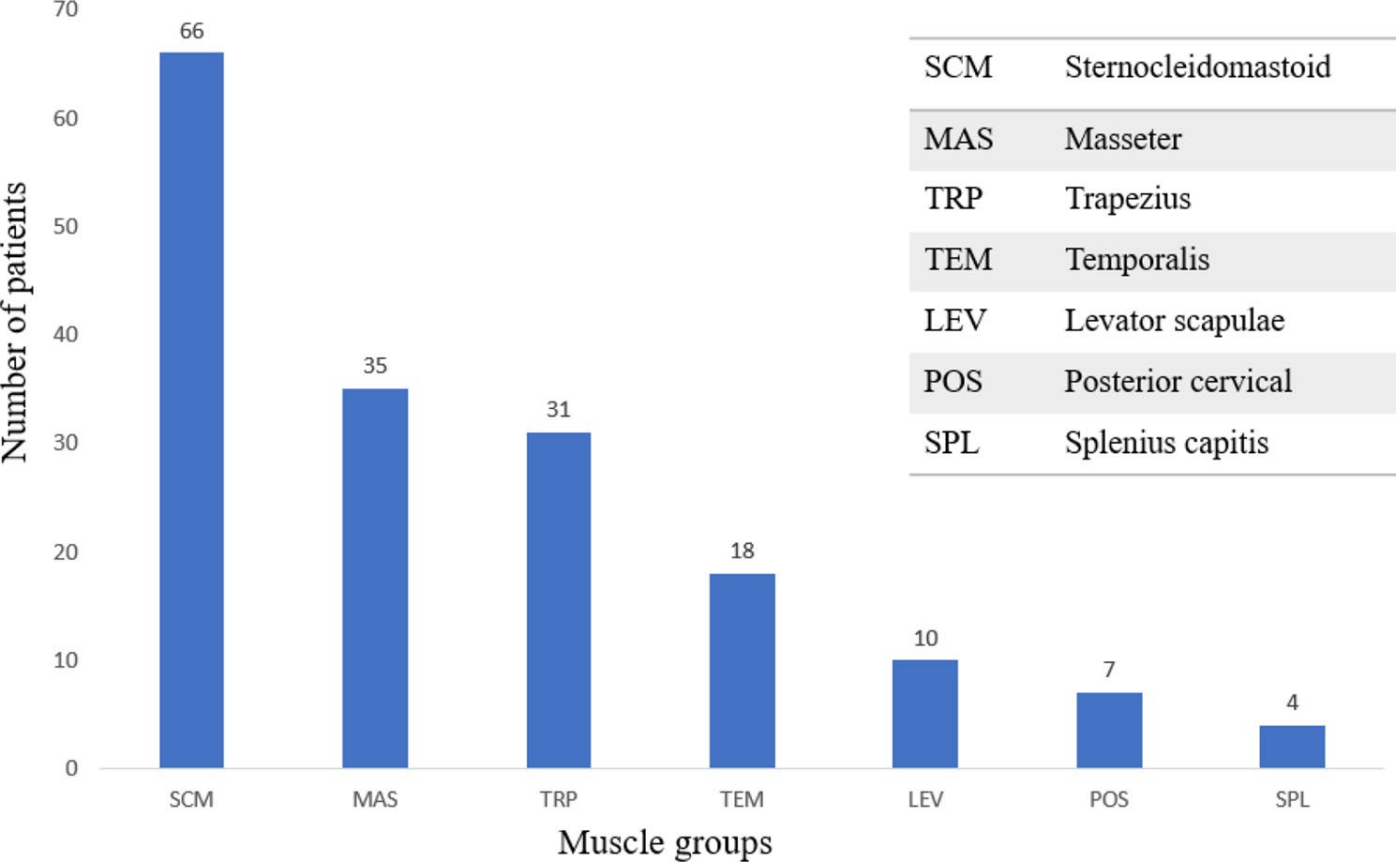
Common muscles involved

**Fig. 2:**
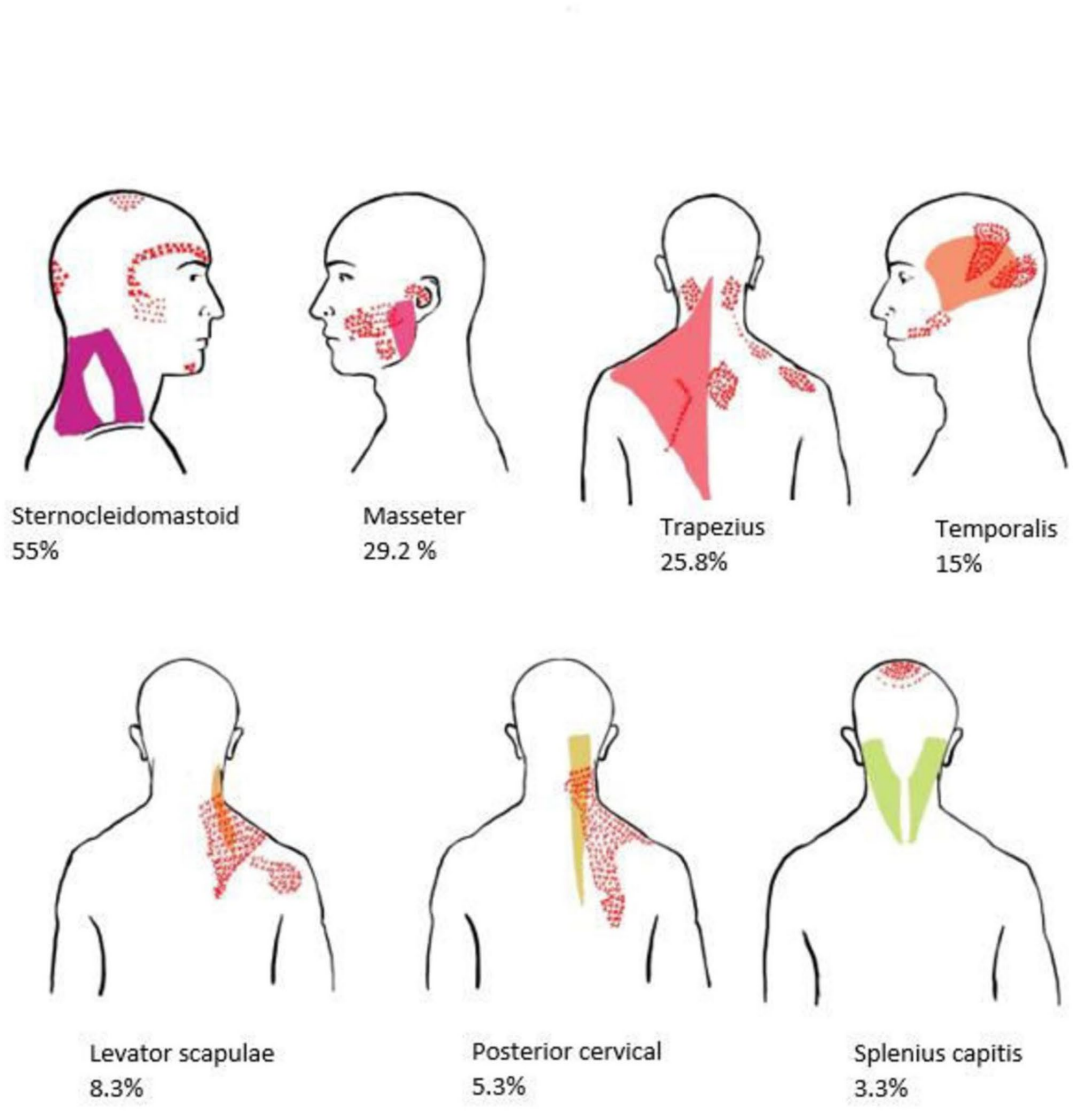
Topographical representation of muscle group involvement

Topography of muscle group involvement indicated that sternocleidomastoid muscle was predominantly involved, in approximately 55.0% of participants, followed by masseter (29.2%), trapezius (25.8%), temporalis (15%), levator scapulae (8.3%), posterior cervical (5.8%), and splenius capitis (3.3%) (Figures 1 and 2). In the study, the distribution of muscle group involvement varied among the patients. Notably, a majority of around 46.7% (*n* = 56) displayed involvement of multiple muscle groups, while 21.7% (*n* = 26) exhibited involvement of a single muscle group.

**Fig. 3:**
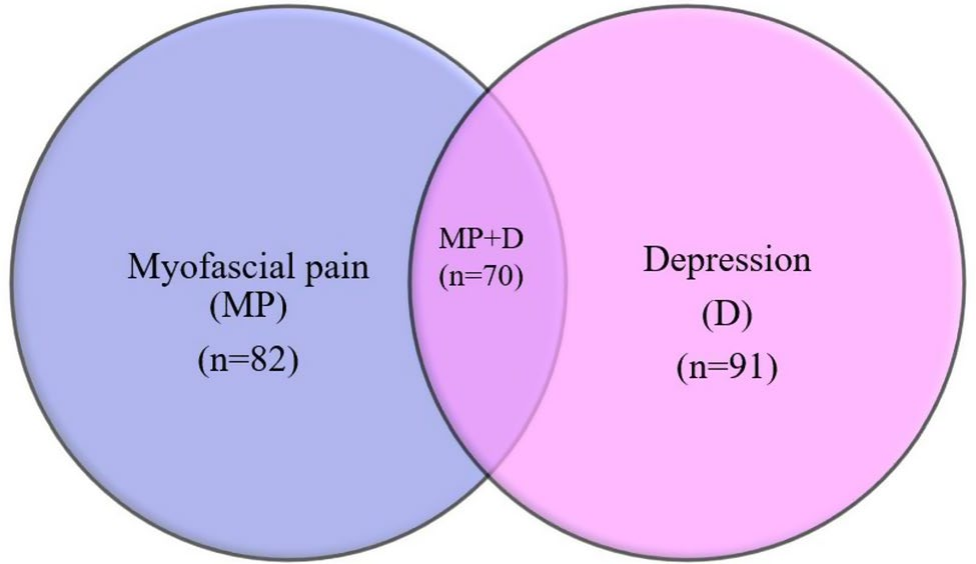
Overlap between myofascial pain and depression

**Fig. 4:**
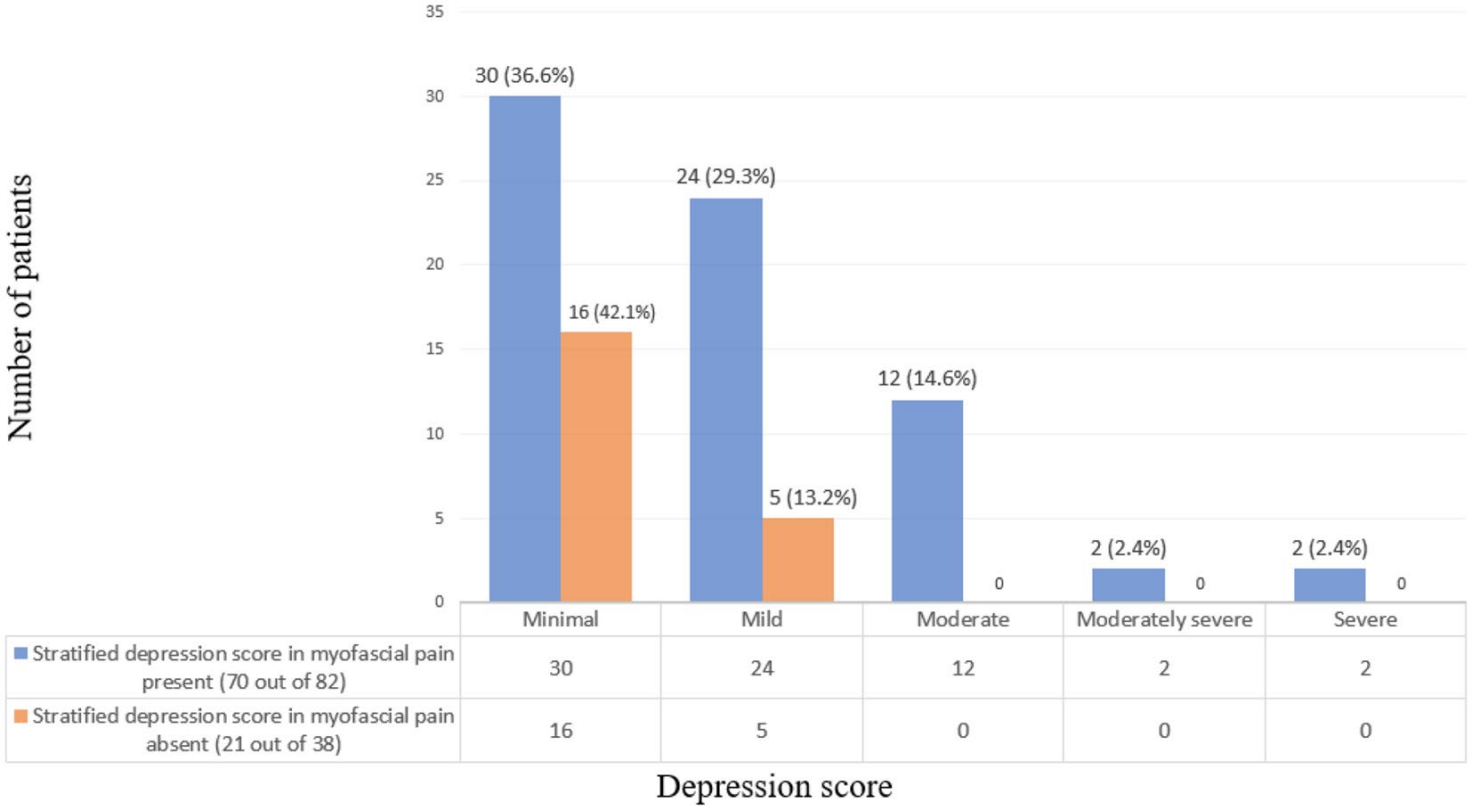
Stratified depression score

PHQ-9 questionnaire was administered to 120 individuals to assess their emotional health in terms of depression, where 75.8% (*n* = 91) scored more than 1 which was suggestive of minimal to severe depression and 24.2% (*n* = 29) scored less than 1. Out of the 82 participants diagnosed with myofascial pain, 85.4% (*n* = 70) individuals had symptoms of depression, whereas the remaining 14.6% (*n* = 12) had no depression (Fig. 3). In the individuals diagnosed with myofascial pain with concurrent depression (*n* = 70), 65.9% (*n* = 54) of the subjects reported experiencing minimal to mild depression, 14.6% (*n* = 12) with moderate, 2.4% (*n* = 2) each with moderately severe and severe depression. Among those patients without myofascial pain (*n* = 38), 55.3% (*n* = 21) exhibited varying levels of depression according to the PHQ-9 depression screening scale which included 42.1% (*n* = 16) with minimal and 13.2% (*n* = 5) with mild depression (Fig. 4). Pain score and depression in patients with myofascial pain were positively correlated with a value of 0.579, (p value < 0.05). In the analysis of mean pain and depression scores across different treatment modalities, including surgery alone (*n* = 2), surgery with adjuvant therapy (*n* = 51), and radiation treatment (*n* = 29), it was observed that depression scores were significantly higher in patients who underwent surgical treatment with a mean score of 11.00, 5.63, 4.76 respectively. However, the mean pain scores were comparable across all treatment modalities, with no significant differences observed between the groups.

The regression coefficients indicate the predicted change in pain score with changes in relevant explanatory variables (age, months post-treatment, and depression score on PHQ9). The overall fit of the model was statistically significant, as indicated by an F-statistic of 16.68 with a p-value less than 0.001, (F (3,78) = 16.68, *p* < 0.001), suggesting that the model explains a significant portion of the variance in pain scores. The adjusted R² value of 0.36 further illustrates that our model can account for approximately 36% of the final pain scores variability, highlighting the included predictors’ substantial impact (Table 2).

## Discussion

In this study, 68.3% of patients treated for head and neck cancer reported experiencing myofascial pain, aligning with existing literature [10]. Kalinchman et al. conducted a narrative review that compiled several randomized controlled trials, noting that the prevalence of myofascial pain in head and neck cancer patients ranged from 11.9 to 44.8%, with variations attributed to differences in diagnostic criteria, patient demographics, and timing of assessments post-treatment [10]. The heterogeneity of myofascial pain syndrome prevalence across studies underscores the need for a standardized approach to diagnosis and evaluation. Additionally, the chronic nature of these symptoms often complicates the clinical picture, as many of the muscle groups affected are involved in the complex anatomical structures of the head and neck region. Our findings regarding the topographical distribution of muscle involvement revealed the sternocleidomastoid muscle as the most affected, followed by the masseter and trapezius muscles. This pattern aligns with the functional role of these muscles in supporting head movement, mastication, and posture, which are often disrupted following extensive cancer treatments like surgery and radiotherapy. Interestingly, previous studies have highlighted different muscle groups, such as the splenius and trapezius, as being predominantly affected [20], indicating potential variability in muscle involvement based on the specific nature of the treatment received, such as the site of radiation or extent of surgical resection. Muscles like suboccipitalis, semispinalis, spinalis and rhomboids are not commonly reported as primary sites of myofascial pain in patients with head and neck cancer. The sternocleidomastoid, masseter, trapezius, temporalis, and levator scapulae are more directly involved in functions affected by head and neck cancer and its treatment, such as chewing, swallowing, neck movement, and postural compensation. The excluded muscles primarily contribute to deeper postural stabilization rather than direct functional impairment in head and neck cancer survivors. They are deeper and more challenging to assess through palpation and clinical examination, making their inclusion less practical. Pain in the deeper paraspinal and scapular muscles may be influenced by generalized postural issues, degenerative spine conditions, or non-head and neck cancer-related musculoskeletal disorders [21]. The cervical and temporomandibular joints are the common sites leading to an exaggerated sensitivity to pain and pressure stimulus in individuals who have survived head and neck cancer [22]. Myofascial pain syndrome in cervical pain is primarily due to mechanical strain, poor posture, or degenerative changes, whereas in cancer, it results from radiation fibrosis, nerve injury, or post-treatment effects. Cancer-related myofascial pain syndrome involves persistent pain, different muscle groups, widespread referral patterns, and often requires multimodal management, including physiotherapy and neuropathic medications [22].

Another critical aspect of the post-treatment burden faced by head and neck cancer survivors, as evidenced in this study, is the psychological distress, particularly depression. Depression in our cohort was notably prevalent, reflecting the considerable emotional challenges faced by this population. This is consistent with earlier studies, which have reported higher levels of anxiety and depression among cancer survivors, with anxiety often peaking during the immediate post-treatment phase [23]. Over time, depression tends to become more pronounced as patients adjust to long-term sequelae such as disfigurement, chronic pain, and functional limitations. The significant decrease in anxiety scores and concurrent increase in depression scores over time observed in other studies may be due to shifting focus from the immediate fear of cancer recurrence to coping with the enduring physical and psychosocial challenges of survivorship [24].

The emotional impact of head and neck cancer and its treatment is serious and needs significant attention. While much attention has been given to survival rates and disease remission, less focus is often placed on the long-term quality of life and emotional well-being of survivors. Our study adds to the growing body of evidence suggesting that depression is a major concern, and emotional well-being should be addressed as part of comprehensive survivorship care. The high prevalence of depression identified through the PHQ-9 depression scale may partly be attributed to the inclusion of somatic symptoms like fatigue, appetite changes, and sleep disturbances, which are common in cancer patients even in the absence of clinical depression. Therefore, future studies should incorporate more nuanced mental health screening tools that distinguish between cancer-related symptoms and psychological disorders. This study emphasizes the importance of early identification and management of both physical and psychological symptoms in head and neck cancer survivors. Early and accurate diagnosis, combined with tailored interventions such as rehabilitation, and multidisciplinary pain management, can significantly mitigate long-term suffering and improve patient outcomes. Psychological interventions should also be integrated into survivorship care plans to address the mental health needs of this vulnerable population.

This study has a few limitations. It is a single-center study that limits the generalizability of our findings to broader populations, as institutional practices in treatment and symptom management may differ. Furthermore, assessments of physical and psychological symptoms were conducted at only one-time point post-treatment, which limits our ability to comment on the progression or resolution of myofascial pain and psychological distress over time. Additionally, the study did not account for pre-existing mental health conditions or differentiate between new-onset depression developed during or after cancer treatment. Screening for mental health disorders at baseline could provide a clearer picture of the mental health burden attributable to cancer treatment.

In this study, physical and psychological aspects of the burden of head and neck cancer treatment are explored. Cancer survivorship after head and neck cancer treatment presents significant challenges, with patients often facing long-term morbidities such as pain, fatigue, disfigurement, and emotional distress. Pain perception reduces physical fitness and functional capacity, and fatigue can significantly impair survivors’ ability to carry out daily activities or maintain employment [14]. A key area for future research is conducting prospective follow-up studies on larger cohorts to explore the trajectory of recovery and the factors that influence successful reintegration into daily life. Understanding these elements could help design targeted interventions to improve quality of life and support patients in achieving functional and psychological recovery.

## Conclusion

Our study found a high prevalence of myofascial pain in patients treated for head and neck cancer with RT and or surgery, especially within the first year of cancer-directed treatment. The muscles involved in neck and shoulder movements were most affected. Many patients had two or more muscle groups involved, impacting daily activities and emotional well-being, including depression. Through this study it highlights the importance of integration of supportive care and symptom management for early identification and management of myofascial pain, thereby improving quality of life post cancer treatment.

## Electronic supplementary material

Below is the link to the electronic supplementary material.

Supplementary Material 1

## Data Availability

No datasets were generated or analysed during the current study.

## Abbreviations

CTRT: Chemotherapy– Radiation therapy
Gy: Gray
PHQ-9: Patient Health Questionnaire-9
RT: Radiation therapy
