# Supplementary material for: Prevalence, patterns, and impact of myofascial pain in patients with head and neck cancer after cancer treatment - a single-center cross-sectional study in India
Source: BMC Palliat Care. 2025 Apr 21;24:109. doi: 10.1186/s12904-025-01745-y (PMC12012951; doi:10.1186/s12904-025-01745-y)

Supplementary file

Appendix no: 1 - Diagnostic criteria for the myofascial trigger points

| **Appendix no: 1 - Diagnostic criteria for the myofascial trigger points** | |
| --- | --- |
| **A. 3 major/essential criteria** | **B. 5 minor criteria** |
| a) Taut band | a) Local twitch response |
| b) Hypersensitive spot | b) Jump sign |
| c) Referred pain | c) Restricted range of motion |
|  | d) Pain with muscle stretching |
|  | e) Pain with muscle contraction |
| **Diagnosis** - At least 2 of the above-mentioned major/essential criteria must be present for trigger point diagnosis. | |

Appendix no: 2 - Patient health questionnaire – 9 (English version)


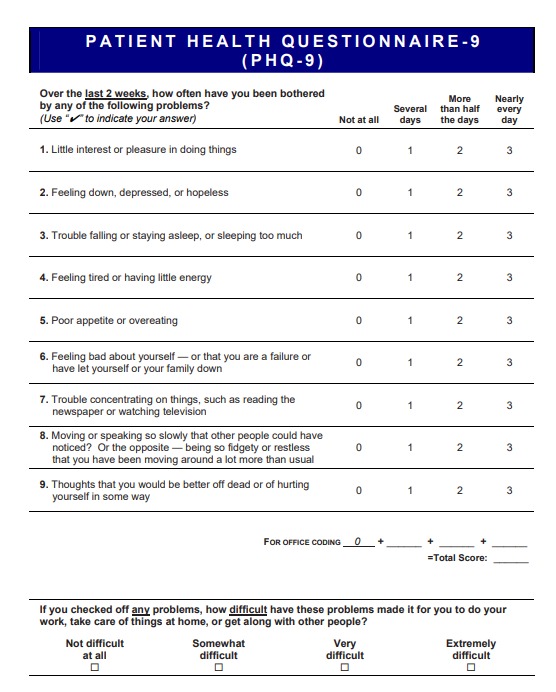


Appendix no: 3 - Patient health questionnaire – 9 (Kannada version)


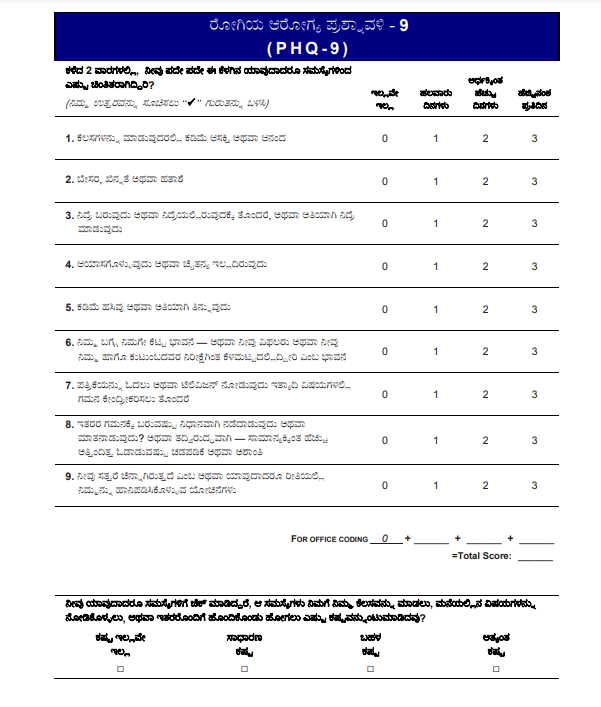

Supplement: Supplementary file 1 — Supplementary Material 1 [file 12904_2025_1745_MOESM1_ESM.docx]
